# Ultrasound in Pediatric Inflammatory Bowel Disease—A Review of the State of the Art and Future Perspectives

**DOI:** 10.3390/children11020156

**Published:** 2024-01-25

**Authors:** André Hoerning, Jörg Jüngert, Gregor Siebenlist, Ferdinand Knieling, Adrian P. Regensburger

**Affiliations:** 1 Department of Pediatrics and Adolescent Medicine, University Hospital Erlangen, Friedrich-Alexander-University (FAU) Erlangen-Nürnberg, 91054 Erlangen, Germany; 2 German Center Immunotherapy (DZI), University Hospital Erlangen, Friedrich-Alexander-University (FAU) Erlangen-Nürnberg, 91054 Erlangen, Germany

**Keywords:** pediatric inflammatory bowel disease, Crohn’s disease, ulcerative colitis, ultrasound, optoacoustics

## Abstract

Inflammatory bowel disease (IBD) comprises a group of relapsing, chronic diseases of the gastrointestinal tract that, in addition to adults, can affect children and adolescents. To detect relapses of inflammation, these patients require close observation, frequent follow-up, and therapeutic adjustments. While reference standard diagnostics include anamnestic factors, laboratory and stool sample assessment, performing specific imaging in children and adolescents is much more challenging than in adults. Endoscopic and classic cross-sectional imaging modalities may be invasive and often require sedation for younger patients. For this reason, intestinal ultrasound (IUS) is becoming increasingly important for the non-invasive assessment of the intestine and its inflammatory affection. In this review, we would like to shed light on the current state of the art and provide an outlook on developments in this field that could potentially spare these patients more invasive follow-up procedures.

## 1. Introduction

Crohn’s disease (CD) and ulcerative colitis (UC) are chronic, relapsing inflammatory conditions of the gastrointestinal tract [1]. The exact etiology and pathogenesis of such inflammatory bowel diseases (IBDs) remain not completely understood [2]. An underlying unregulated inflammatory immune response and interaction with the intestinal microbiota in genetically predisposed individuals is hypothesized [3,4]. Exogenous environmental influences and the composition of the intestinal microbiome also play a role in disease development [5]. IBDs in children and adolescents often have unusual manifestations, are exhibited more frequently, and are usually more severe and more difficult to treat [6]. Disease progression occurs in 31 or 49% of pediatric patients with CD or UC, respectively, while stable localization is observed in 90 or 80% of patients with first manifestation in adulthood.

For example, the increase in activity during the first years of the disease, the need for immunosuppressive therapy, and the need for surgery are significantly higher in pediatric IBD than in adult patients [6].

CD can be localized throughout the gastrointestinal (GI) tract and is characterized by segmental, discontinuous involvement and inflammatory changes affecting all layers of the intestinal wall [7]. Initial clinical symptoms are often nonspecific and include abdominal pain, usually bloodless diarrhea, weight loss, and fever. Especially in children, an appendicitis-like clinical picture may present if the terminal ileum is affected. More commonly than in adults, 51% of pediatric CD patients show an affection of the upper gastrointestinal tract, with the anatomic extent increasing in 39% of patients within 2 years [8].

In contrast, UC affects the distal rectum and continuously spreads orally. The clinical symptoms are often bloody, mucopurulent diarrhea accompanied by fever and abdominal pain, which often occurs as tenesmus in the left lower abdomen before defecation [9]. Symptoms ranging from bleeding with iron deficiency and anemia to massive blood loss and a dilated toxic megacolon with risk of developing peritonitis, sepsis, and perforation are among the major intestinal complications [10]. In childhood, about two thirds of patients display pancolitis upon manifestation of UC [11]. Macroscopically, the clinical picture is characterized by extensive redness and swelling of the mucosa with contact hemorrhages and, during the course of the disease, the formation of inflammatory pseudopolyps. Besides affecting intestinal organs, CD and UC may present with extraintestinal manifestations including affection of the skin, eyes, joints, and liver. Such manifestations include erythema nodosum, conjunctivitis, arthritis, and primary sclerosing cholangitis (PSC) [12].

The current therapeutic strategies include modulation of the immune system and targeting of major cytokine pathways [13,14]. Some examples are blocking agents of pro-inflammatory tumor necrosis factor alpha (TNFα) [15], leucocyte adhesion molecules such as α4β7-Integrin or signal kinases [16], and inhibitor of T-cell Janus kinases (JAKs) [17].

In both Western industrialized and developing countries, an increase in the prevalence and incidence of IBD has been observed in recent decades [18,19,20]. This trend can also be observed in pediatric patients, along with an increasingly early onset of disease in this group [11,21]. In approximately 20–30% of all affected patients, IBD manifests in childhood [11]. In Germany, the incidence of CD is estimated to be 6.6 per 100,000 habitants, with a prevalence of approximately 100–200, while the incidence of UC is reported to be 3.0–3.9, with a prevalence at approximately 160–250 [22]. The diagnosis of IBD is based on a synopsis of history, clinical examination findings, imaging results, endoscopy with histology, and laboratory values (so-called Porto diagnostic criteria) [23,24,25]. Generally, the incidence of IBDs may increase with age, and the peak incidence is around 14 to 15 years, as reported in a large European cohort [26]. As intestinal ultrasound (IUS) is an emerging non-invasive point-of-care tool for accurately detecting and monitoring disease activity [27,28,29,30,31], this narrative review article will provide current state-of-the-art and novel developments in the field of non-invasive imaging of the intestine in pediatric IBD.

## 2. Diagnostic Imaging and Surveillance Approaches in Pediatric IBD

IBDs are often challenging, both from a diagnostic and therapeutic point of view. While deep-seated anatomic structures are difficult to assess via US alone, diagnostic imaging also includes magnetic resonance imaging (MRI). Commonly, oral mannitol administration is used—termed magnetic resonance enterography (MRE)—in order to better visualize the small intestine [23] and pelvic MRI to assess anal fistulas or perianal abscesses. However, the evaluation of the upper GI-tract (stomach and duodenum) is difficult via IUS. In comparison to US where data is scarce, MRE shows good performance in detecting IBD in pediatric patients [32]. A meta-analysis in 687 patients demonstrated a sensitivity of 83% and specificity of 93% for the detection of active inflammation with known or suspected IBD [33]. Therefore, MRI/MRE has its value in establishing the diagnosis and providing support in distinguishing between CD and UC. Likewise, gastrointestinal endoscopy is invasive, expensive, and time-consuming. In contrast, US is safe, fast, and cheap, and does not require any anesthesia, while it can be used both for monitoring children with IBD and for children suspected of having IBD [34]. With respect to very young patients (very early onset, VEO-IBD), the European Society of Paediatric Radiology abdominal imaging task force recommends the first-line use of US [35]. MRE is used for further work-ups in the case of unclear US findings, while the use of computed tomography techniques is limited to specific situations. In the adult IBD context, gastrointestinal endoscopy and MRE are the gold standard tests to establish a diagnosis for IBD (Porto criteria) [36]. Consequently, the evaluation and monitoring of disease activity may be complemented by using US.

Intestinal US is teachable to healthcare physicians in a training curriculum for instance, and is then able to bring at least moderate diagnostic accuracy while not having to be exclusively performed by experienced radiologists [37]. However, there is a lack of consensus on how a specific training curriculum for children should look. Moreover, US is generally well accepted by pediatric patients, and therefore, their preferred diagnostic modality [38,39].

## 3. US in Pediatric Inflammatory Bowel Disease

### 3.1. US Anatomy of the Intestinal Wall

**Figure 1:**
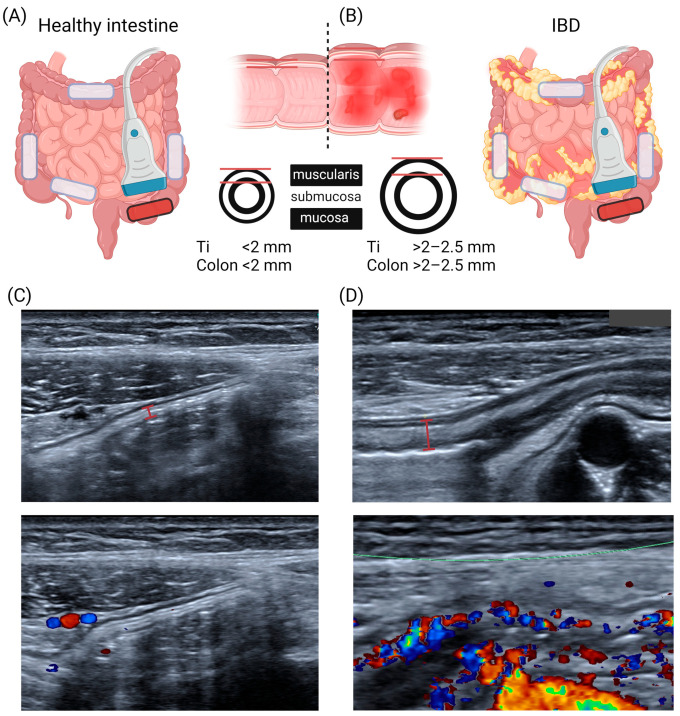
Ultrasound for assessment of inflammatory bowel diseases (**A**,**B**) Schematic cartoon of standard imaging locations for disease activity assessment in inflammatory bowel diseases as follows: terminal ileum and ascending, transverse, descending, and sigmoid colon. In healthy subjects, the intestinal wall of the terminal ileum/colon is thinner than 2 mm and it exceeds 2–2.5 mm in inflamed segments in IBD patients. (**C**,**D**) Exemplary B-mode and color Doppler images of healthy and inflamed segments of the sigmoid colon with exemplary bowel wall measurements. Displayed is a healthy child and one pediatric patient with UC. B-mode images show enlarged submucosa in the UC patient with increased blood flow measured by color Doppler. IBD = inflammatory bowel disease, Ti = terminal ileum. Created with https://www.biorender.com/ (accessed on 22 January 2024).

Intestinal US, especially in pediatrics, is performed with frequency transducers, typically in the range of 7.5–17 MHz. In almost all cases, supine positioning of the patient is recommended [40]. This way, the intestinal wall presents with a typical layered anatomy (Figure 1, Table 1) [41]. In addition, ultrasound as a dynamic examination provides important information in real time on the dynamics of intestinal activity in terms of peristalsis and lumen width. In addition, color Doppler enables the visualization of increased perfusion in inflamed segments of the intestine.

Knowledge of regular US anatomy serves as the baseline for physicians in order to use such technologies in IBD diagnostics.

### 3.2. Current US Information Used in IBD Diagnostics

While endoscopic procedures are generally restricted to the evaluation of the mucosal surface, and histological assessments inevitably take days, abdominal US is more than an adjunct tool in order to assess the inflammation of the intestinal wall [42]. US examinations can immediately provide information on concomitant mesenterial lymphadenopathy and bowel wall thickness (BWT), while Doppler signals are used as surrogates of intestinal blood flow changes and the detection of mesenteric inflammatory fat. Together, these alterations can help in interpreting the extent of severity and in the prediction of disease activity in IBD. The ultrasonographic appearance of Crohn’s disease is characterized by segmental inflammation, asymmetric thickening of the intestinal wall, and transmural inflammation, as depicted by increased perfusion [34].

In cases of long-standing disease, IUS is able to provide additional information, as untreated or insufficiently treated IBD can result in an extensive and circumferential loss of the characteristic tissue layers of the intestinal wall, appearing sonographically as a hypoechoic rim with abrogated stratification. As a result of the fibrofatty alteration of the mesenteric tissue, affected segments tend to be contrasted and fixed without peristalsis with the impression of an abnormal angulation [41]. In cases of chronic inflammatory, active disease, these features may be additionally associated with luminal stenosis, rigid and immobile bowel segments, or even loss of the haustra, e.g., in the colon [41]. Further complications are the development of abscess and/or fistula formations or a mechanical ileus due to pronounced luminal stenosis.

In general, studies using the conventional B-mode US in Crohn’s disease to detect inflammatory lesions at the terminal ileum demonstrated an overall sensitivity of 74–88%, and an overall specificity of 78–93% [43]. For instance, when Canani et al. assessed the effectiveness of ultrasonographic bowel wall measurement in the diagnostic work-up of children with suspected IBD by combining it with established and, to some extent, distinctive laboratory markers, such as the determination of fecal calprotectin, anti-Saccharomyces cerevisiae antibodies, and perinuclear staining antineutrophil antibodies [44], they found that UC-directed bowel wall measurement proved to be an accurate, non-invasive, and reproducible technique for the detection of inflammation localized in the ileum. Their results even led to the interpretation that abdominal US may be used as a primary imaging procedure in children with suspected IBD [34].

However, with respect to the above-described characteristic, but mainly qualitative assessments of alterations of the intestinal bowel and its surrounding anatomical structures, there is a great need for a standardization of the use of US in pediatric IBD patients. To achieve a standardized examination procedure in children, we must defined which criteria in quality and quantity should be taken into account to define an abnormal US in the first place. This poses an important unmet prerequisite, since a recent systematic review on the diagnostic accuracy of IUS showed that to date, no common criteria to define an IUS as abnormal are in use [45]. More interestingly, in adults, there is a so-called expert consensus on the optimal acquisition and development of the International Bowel Ultrasound Segmental Activity Score (IBUS-SAS), identifying four major parameters: bowel wall thickness (BWT); bowel wall stratification; hyperemia of the wall (color Doppler imaging), and inflammatory mesenteric fat [46].

### 3.3. Bowel Wall Thickness (BWT)

A meta-analysis reported bowel wall thickness values ranging from 0.8 to 1.9 mm in the small bowel and from 1.0 to 1.9 mm in the colon with increasing with age (Table 2) [47].

This means that BWT assessed via US is mostly reported to be 1.2 mm on average and does not reach values above 2 mm [48,49,50]. In the last twenty years, only a few studies have dealt with the differences in BWT between different categories of disease severity [45]. In contrast to normal measures, the mean value for BWT was 1.7 ± 0.4 mm in remission, 2.4 ± 0.4 in mild, 3.5 ± 0.5 in moderate, and 4.8 ± 0.7 in severe endoscopic disease activity, respectively. In adults, transmural remission in both CD and UC was defined by bowel thickness ≤ 3 mm, and to assess treatment response, a reduction in BWT of over 25% or over 2.0 mm or over 1.0 mm and one color Doppler signal reduction were defined [31]. With regard to such data, the optimal cut-off may be defined as 2–2.5 mm. The findings of another study from Chioran and co-workers demonstrated that children with Crohn’s disease exhibited an increased thickness of the ileocecal intestinal wall (>3 mm) when compared to healthy age-matched subjects (less than 2 mm) [51].

Increased BWT in the presence of hyperemia is frequently seen in both subtypes of IBD. It is thus not always possible to clearly distinguish UC from CD using the B-mode alone [30]. Voogd et al. reported BWT to be an accurate parameter for monitoring treatment response in adult patients receiving tofacitinib treatment [52]. Whether or not this is applicable in younger patients remains to be determined. However, these data require confirmation in future prospective multicenter studies with respect to subtypes, treatment strategies, and individual courses of the disease. Additionally, there is still a requirement to define the exact methodology for measuring BWT, for example, on longitudinal or cross-sectional images (see Figure 1).

### 3.4. US Doppler Signals

Besides BWT, the very first approaches using ultrasound to diagnose and monitor inflammatory activity in IBD also assessed Doppler signals in the bowel wall. Limberg et al. used a qualitative, descriptive approach to grade scores from I to IV (Table 3) [53].

For example, such measurements of vessel density were able to reflect disease activity in patients with CD [54]. In 2004, Scholbach et al. reported flow velocities inside intestinal walls derived from recorded Doppler ultrasounds in 34 healthy children and 14 pediatric patients with CD [55]. In the small intestine, the flow velocity was 0.025 cm/s in healthy participants and elevated to 0.095 cm/s in those with CD, and in the large intestine, these values were 0.012 cm/s vs. 0.082 cm/s, without a strong correlation of clinical activity indices [55]. On a microscopic scale, preclinical studies have found that despite an increase in volumetric flow during inflammation, the actual velocity in the smallest intestinal capillaries decreased to create conditions suitable for leucocyte adhesion and transmigration [56]. With new technological developments, such as ultrasensitive Doppler, these criteria will also be adapted for children.

### 3.5. Mesenterial or “Creeping Fat”

A feature of CD is the extra-intestinal appearance and expansion of so called “creeping fat”, which may prevent (together with fibrosis) the systemic translocation of gut bacteria [57]. Interestingly, this “organ” is not a passive bystander in intestinal inflammation and might harbor two—harmful and beneficial—sites in this regard; while it might develop as a reaction to intestinal injury, leading to limited bacterial dissemination, it does not show a switch-off in CD [57,58,59]. It is now well understood that adipose tissue is associated with major alterations in the secretion of cytokines and adipokines, which mediate the immune-metabolic crosstalk of immune, lymphatic, neuroendocrine, and intestinal epithelial systems in IBD [60]. Calculating the slope of the Hounsfield unit (HU) curve of “creeping fat” on energy spectral computed tomography (CT) images, it could be shown that this correlated with endoscopic (SES-CD, r = 0.66, *p* < 0.01) and clinical disease activity (Harvey-Bradshaw index, r = 0.414, *p* < 0.01) [61]. A novel mesenteric creeping fat index (MCFI) has been shown to accurately characterize the extent of mesenteric fat wrapping in surgical specimens [62]. This might be particularly important in planning surgery for CD, because the inclusion of the mesentery in ileocolic resections may alter the course of CD [63,64], reducing the risk for recurrence requiring reoperation [65]. “Creeping fat” can be assessed in US and may also correlate with inflammatory activity [66]. So far, it has been used far less as a single characteristic for measuring clinical outcomes in IBD but more frequently in multiparametric intestinal US scoring systems. Furthermore, more specific studies in children and adolescents are missing.

### 3.6. Fibrostenosis and Intestinal Strictures

Fibrostenosis is the permanent and abnormal deposition of extracellular matrix (ECM, primarily collagens) to the intestinal wall, leading to a narrowed lumen with proximal dilation [67]. This process is still often regarded as irreversible following long-term inflammation in patients with less favorable responses to therapy [68]. The most important predilection site for such complications in patients with CD is the terminal ileum [69]. Although it appears to be more common in CD, similar complications can also occur in UC. It can already occur at the time of diagnosis, with rates of up to 21% in patients with CD and 1–11% in patients with UC [70]. However, an optimal anti-inflammatory regime including early anti-TNFα therapy did reduce the risk of penetrating but not fibrostenoic complications in children [71]. This, in turn, calls into question the effectiveness of current anti-inflammatory therapies in preventing such complications.

Various signaling pathways, growth factors, and cytokines, including IL-13, platelet-derived growth factor (PDGF), connective tissue growth factor, basic fibroblast growth factor, insulin-like growth factor, bone morphogenetic proteins (BMPs), and transforming growth factor-β (TGFβ), have been associated with its development [67,72]. According to Kugathasan et al., there might be an ECM gene signature that can predict the development of stricturing complications [71]. For this reason, it would be highly desirable to be able to reliably detect these processes at an early stage using non-invasive US, and also to identify patients at risk for unfavorable outcomes. More specifically, given the high frequency of intestinal strictures, the differentiation between fibrotic and inflammatory strictures might be crucial for clinical decision making [73].

A systematic review including 14 studies (511 adult subjects) found that US can currently not differentiate between fibrotic and inflammatory stenosis in CD patients [74]. Given the idea that these intestinal sections were subject to remodeling over a longer period of time, the main parameters used were B-mode US, strain elastography, shear wave elastography, and contrast-enhanced ultrasound (CEUS) [75,76,77,78,79,80,81,82,83,84,85,86,87,88,89,90,91,92]. These are primarily intended to demonstrate the altered intestinal wall structure or stratification with abnormal deposition of ECM, vascular changes, and an increase in stiffness. A first study with CEUS in pediatric IBD containing a small number of patients (*n* = 25) shows encouraging results [93], but these protocols are far from standardization or routine use. In the future, such techniques could become more sophisticated may be able to better capture changes in flow behavior in the tissues of smaller children [94,95,96] or even depict and quantify small microstructures [97].

### 3.7. US Scoring Systems

In addition to the aforementioned imaging features, ultrasonographic activity indices or scores would definitely be desirable and represent an unmet need. Concerning the latter, the field is moving forward as there have been a couple of promising approaches (Table 4).

Civitelli et al. introduced a prospectively studied score for children with UC combining five US items [98]. These included bowel wall thickness (>3 mm), bowel wall stratification, vascularity, the presence of haustra coli, and enlarged mesenteric lymph nodes (51). Similarly, Wassenaer et al. investigated pediatric UC, reporting the so-called ulcerative colitis intestinal ultrasound (UC-IUS) index [99]. UC-IUS performed better than the Civitelli score with respect to the endoscopic subscore [102]. The pediatric CD intestinal US (PCD-US) index was developed to assess disease activity more specifically in CD [100]. The PCD-US index was evaluated in a prospective study, wherein the assessment was validated with the simple endoscopic score for CD (SES-CD) [103]. Kellar et al. developed their Simple Pediatric Activity Ultrasound Score (SPAUSS) for both IBD subtypes in a small retrospective study, dealing with four US parameters, including bowel wall thickness (BWT), mesenteric inflammatory fat, lymphadenopathy, and hyperemia [101]. Here, more emphasis was put on the presence or absence of mesenteric inflammatory fat to predict disease severity. A major limitation lies in the fact that SPAUSS has unfortunately not been validated against an endoscopic score.

Recently, a blinded, cross-sectional cohort study in young CD patients demonstrated the high sensitivity and specificity of IUS scores and BWT compared with the endoscopic SES-CD score [29]. Such scores or indices may correctly reflect endoscopic disease activity [104], but further external validation in prospective multicenter approaches is recommended to accelerate integration into clinical guidelines and clinical practice.

## 4. Novel US-Based Imaging Technologies: Optoacoustic Imaging (OAI)

While conventional US is based on the piezoelectric effect, discovered by the Curie brothers in 1880 [105], optoacoustic imaging is a further development of the photoacoustic effect, discovered by Alexander Graham Bell in the same year [106]. In contrast to US, OAI was applied in humans much later, with first descriptions in the early 1990s [107,108]. In OAI, light is used to induce the movement of molecules in deep tissue, and the scattered ultrasound waves are then detected [109,110]. By applying several wavelengths, specific optoacoustic spectra of different endogenous (oxygenated and deoxygenated hemoglobin, lipids, collagens, and melanin) and exogenous chromophores (dyes like indocyanine green) can be detected and quantified [111,112,113]. Hemoglobin is especially predestined for OAI, as it is one of the main absorbers in the used Near-infrared field of light and a surrogate OAI biomarker for various inflammatory [114] and cardiovascular diseases [115,116,117]. Furthermore, OAI allows scalability of the imaging device and resolution from cells and animal models to humans [110].

**Figure 2:**
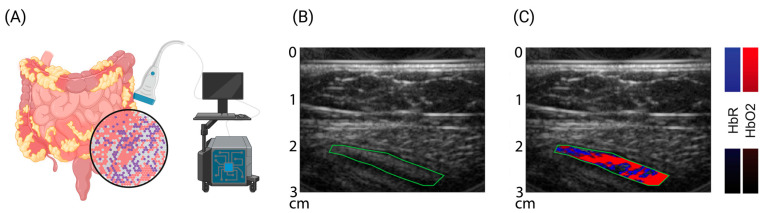
Multispectral optoacoustic imaging—molecular sensitive ultrasound. (**A**) Cartoon of bedside multispectral optoacoustic tomography (MSOT) to assess molecular tissue composition. (**B**,**C**) Exemplary reflected-ultrasound computed tomography (RUCT) and MSOT images of a pediatric patient with ulcerative colitis. RUCT enables real-time guidance of the investigator. Spectral unmixing of MSOT data allows differentiation and quantification of oxygenated (red) and deoxygenated (blue) blood within the bowel wall (green, region of interest). Increased levels of hemoglobin correlate with disease activity and might serve as surrogate biomarkers. Created with https://www.biorender.com/ (accessed on 22 January 2024).

In murine models of colitis, disease severity can be measured by means of increased signal intensities for hemoglobin transabdominally in vivo [118,119], and by using transrectal guidance, changes in the intestinal wall thickness and vessel architecture can even be detected [120]. Furthermore, OAI technologies have the potential to identify intestinal inflammation and fibrosis to characterize intestinal strictures in mice [121,122]. The first pilot studies in adult patients with Crohn’s disease used a handheld OAI system to carry out so-called multispectral optoacoustic tomography (MSOT) to assess disease activity in comparison to clinical, laboratory, endoscopic, and conventional ultrasound [123,124]. The MSOT signal levels for hemoglobin correlated well with disease phenotype with minimal effort from the patient, and with high accuracy in the detection of remission and active disease [124]. In addition, studies on the precision of MSOT for imaging the human intestine showed resilient data [125], and the option of imaging luminal contrast agents throughout the intestinal tract opens the door to further translational applications and functional assessment of the gastrointestinal tract [126,127,128]. Such approaches might help to delineate complications likes fistulae, which are common manifestations in CD patients [129,130]. Early pilot studies in other pediatric conditions (neuromuscular disorders) were promising [131,132,133], and the first study in pediatric patients with UC and CD confirmed previous findings in adults [134] (Figure 2).

Currently, the multicenter approval study of MSOT in adult patients with Crohn’s disease is closed (https://euphoria2020.eu/, accessed on 22 January 2024), and further longitudinal studies in pediatric IBD are expected. For the manifestation of Crohn’s disease in the upper gastrointestinal tract, the integration of OAI in (capsule) endoscopic devices might allow the molecular assessment of the disease similarly to prior studies of the esophagus [135,136,137].

Therefore, OAI might aid in the bedside assessment of molecular disease activity and remission both in adult and pediatric IBD.

## 5. Conclusions

Intestinal US has undergone significant development in recent years. While technical progress and standardized examination methods have been introduced in the field of adult medicine in particular, some of these developments are still pending in pediatrics. The entire field still lacks prospective, multicenter studies that exploit the usability of IUS, particularly with regard to disease and therapy monitoring. However, from the perspective of a physician, the implementation of basic US categorization into the clinical routine follow-up procedure for IBD should rely on the quantitative assessment of the intestinal wall thickness. Hence, an intestinal wall that exceeds 2–3 mm in inflamed segments with increased blood flow should alert doctors to possible IBD lesions or a flare in already diagnosed patients.

This review is limited by its narrative character. Systematic reviews are necessary to determine standard examinations, cut-off values and scoring procedures. However, this review provides important information to perform pediatric IUS and integrate this into future clinical studies. Furthermore, emerging optoacoustic technologies are introduced to the readership to highlight new methods of non-invasive disease assessment in IBD. The molecular decryption and quantification of tissue composition, labeling of targeted therapies, and mapping of microvasculature will pave the way for the assessment and monitoring of individualized precision medicine.

The great advantages of IUS compared to other diagnostics in pediatric IBD are the non-invasiveness, the broad availability, the low infrastructure costs, and the easy-to-learn imaging modality with simple comprehensibility. For routine IUS diagnostics in clinical practice, at least the BWT and Limberg Score might be assessed. And, from our perspective, IUS should be implemented as a clinical endpoint in future clinical trials. However, more clinical studies [138] and robust scores with this methodology are needed to routinely implement IUS as a primary endpoint in clinical trials [139], like in the STARDUST study [140].

In the future, technological improvements and new technologies could provide a large number of other imaging biomarkers, making US-based imaging the first-line diagnostic method in pediatric IBD imaging.

## Figures and Tables

**Table 1:** Ultrasound anatomy of the intestinal wall (from inside to outside layers), modified from Strobel, Goertz, and Bernatik [41].

US Aspect	Anatomic Structure	Microscopic Aspect/Tissue Composition
Hypoechoic (fluid) or hyperechoic (air) lumen		Intestinal content (stool, air, fluids)
Hyperechoic entrance	Transition lumen/mucosa	
Hypoechoic	Mucosa	Epithelial cells
Hyperechoic	Submucosa	Connective tissue
Hypoechoic	Muscularis propria	Muscle cells
Hyperechoic	Transition muscularis propria/serosa, surrounding structures (fat, peritoneal wall)	Epithelial cells, connective tissue, fat

**Table 2:** Pooled mean bowel wall thickness modified from van Wassenaer et al. [47].

Age (Years)	Jejunum (mm)	Ileum (mm)	Cecum (mm)	Asc. Colon (mm)	Tr. Colon (mm)	Desc. Colon (mm)	Jejunum (mm)
0–4	1.0 ± 0.4	1.3 ± 0.6	1.1 ± 0.2	1.1 ± 0.2	1.0 ± 0.2	1.1 ± 0.2	1.0 ± 0.4
5–9	0.8 ± 0.1	0.9 ± 0.1	1.1 ± 0.1	1.1 ± 0.2	1.2 ± 0.2	1.2 ± 0.2	0.8 ± 0.1
10–14	0.8 ± 0.1	1.0 ± 0.2	1.4 ± 0.2	1.3 ± 0.3	1.3 ± 0.2	1.3 ± 0.2	0.8 ± 0.1
15–19	0.9 ± 0.1	1.1 ± 0.1	1.6 ± 0.2	1.4 ± 0.2	1.4 ± 0.2	1.4 ± 0.2	0.9 ± 0.1

**Table 3:** Ultrasound (doppler) scoring of inflammatory activity according to Limberg [53].

Grade	B-Mode	Doppler
Limberg I	Intestinal wall thickening (hypoechoic, sometimes hyperechoic submucosa, partial loss layers)	No intramural vessels
Limberg II	Intestinal wall thickening	Short-stretched vessels detectable
Limberg III	Intestinal wall thickening (homogenous, hypoechoic)	long-stretched vessels detectable
Limberg IV	Intestinal wall thickening	Long-stretched vessels detectable reaching the adjacent mesentery

**Table 4:** Overview of pediatric sonographic US indices.

Reference	Name of Scoring System	Disease	N	Items	Measures of Accuracy
[98]	Civitelli	UC	60	Bowel wall thickness > 3 mm, bowel wall stratification, vascularity, presence of haustra coli, and enlarged mesenteric lymph nodes.	90% concordance with endoscopy (95% CI: 0.82–0.96)
[99]	UC-IUS	UC	35	Bowel wall thickness, Doppler signals, colonic haustrations, wall layer stratification, presence of mesenteric fat wrapping	AUROC for detecting Mayo endoscopic score ≥ 2 Asc. Colon: 0.82 Trans. Colon: 0.88 Desc. Colon: 0.84
[100]	PCD-US	CD	74	Bowel wall thickness, bowel wall perfusion, mesenteric fat proliferation, visibility of colonic haustrations, visibility of wall layer stratification, peristalsis, presence and size of lymph nodes, presence of complications	AUROC for detecting inflammation Terminal ileum: 0.73 Colon: 0.75
[101]	SPAUSS	IBD	75	Bowel wall thickness (BWT), mesenteric inflammatory fat, lymphadenopathy, and hyperemia	AUCROC distinguish active disease from normal condition (absence of disease): 0.82

UC = ulcerative colitis, CD = Crohn’s disease, IBD = inflammatory bowel disease, AUROC = area under the receiver operating characteristic curve, UC-IUS = ulcerative colitis intestinal ultrasound, PCD-US = pediatric Crohn’s disease intestinal ultrasound, SPAUSS = Simple Pediatric Activity Ultrasound Score.

## Data Availability

Not applicable.
